# Routine application of the Lymph2Cx assay for the subclassification of aggressive B-cell lymphoma: report of a prospective real-world series

**DOI:** 10.1007/s00428-022-03420-6

**Published:** 2022-10-11

**Authors:** Alberto Zamò, Elena Gerhard-Hartmann, German Ott, Ioannis Anagnostopoulos, David W. Scott, Andreas Rosenwald, Hilka Rauert-Wunderlich

**Affiliations:** 1 Institute of Pathology, University of Würzburg, Würzburg, Germany; 2 Comprehensive Cancer Center Mainfranken, University Hospital Würzburg, Würzburg, Germany; 3 Department of Clinical Pathology, Robert-Bosch-Krankenhaus, and Dr. Margarete Fischer-Bosch-Institute of Clinical Pharmacology, Stuttgart, Germany; 4 Centre for Lymphoid Cancer, BC Cancer, Vancouver, Canada

**Keywords:** Diffuse large B-cell lymphoma, Hans algorithm, Lymph2Cx assay, Cell of origin

## Abstract

The subclassification of diffuse large B-cell lymphoma (DLBCL) into germinal center B-cell-like (GCB) and activated B-cell-like (ABC) subtypes has become mandatory in the 2017 update of the WHO classification of lymphoid neoplasms and will continue to be used in the WHO 5^th^ edition. The RNA-based Lymph2Cx assay has been validated as a reliable surrogate of high-throughput gene expression profiling assays for distinguishing between GCB and ABC DLBCL and provides reliable results from formalin-fixed, paraffin-embedded (FFPE) material. This test has been previously used in clinical trials, but experience from real-world routine application is rare. We routinely applied the Lymph2Cx assay to day-to-day diagnostics on a series of 147 aggressive B-cell lymphoma cases and correlated our results with the immunohistochemical subclassification using the Hans algorithm and fluorescence in situ hybridization findings using break-apart probes for *MYC*, *BCL2*, and *BCL6*. The routine use of the Lymph2Cx assay had a high technical success rate (94.6%) with a low rate of failure due to poor material and/or RNA quality. The Lymph2Cx assay was discordant with the Hans algorithm in 18% (23 of 128 cases). Discordant cases were mainly classified as GCB by the Hans algorithm and as ABC by Lymph2Cx (n = 11, 8.6%). Only 5 cases (3.9%) were classified as non-GCB by the Hans algorithm and as GCB by Lymph2Cx. Additionally, 5.5% of cases (n = 7) were left unclassified by Lymph2Cx, whereas they were defined as GCB (n = 4) or non-GCB (n = 3) by the Hans algorithm. Our data support the routine applicability of the Lymph2Cx assay.

**Supplementary Information:**

The online version contains supplementary material available at 10.1007/s00428-022-03420-6.

## Introduction

The diagnostic entity of diffuse large B-cell lymphoma (DLBCL) was introduced in the 1994 REAL classification [1] as an effort to unify the several morphologic/immunologic variants of the Kiel classification and the clinically oriented approach of the Working Formulation classification. It was already acknowledged at that time that DLBCL probably comprised more than one biological entity, as reflected by the variable morphology and clinical behavior, but reproducible methods to distinguish them were not available. High-throughput gene expression profiling (GEP) became available in the late 1990s as a powerful method to analyze and quantify several thousand RNA transcripts in a highly parallel way, thus allowing the determination of disease-specific RNA signatures and the discovery of novel biologically defined disease entities by unsupervised clustering methods. This powerful technology was applied to DLBCL [2, 3] and demonstrated that DLBCL comprised at least two robust clusters, which showed either an expression signature similar to purified germinal center B-cells (the GCB-like group) or an in vitro activated B-cell (the ABC-like group). In addition, a group of cases was left unclassified by this algorithm, pointing to the probable presence of further sub-entities. Despite the later definition of other DLBCL subgroups by GEP [4–7], the ABC-GCB signature remained the most accepted way of subclassifying DLBCL, because it bore a significant clinical impact, with patients with an ABC-type disease faring worse than those in the GCB group [8], and predicted responses to specific therapies [9–11]. Based on the reproducible prognostic impact, the distinction between ABC and GCB categories became mandatory in the 2017 classification of lymphoid neoplasms [12]. Due to technical limitations and costs, the 2017 blue book allowed usage of surrogate technologies for the subclassification, which in most cases take advantage of one of the several immunohistochemical classifiers, most commonly Hans’ [13]. Immunohistochemical subclassification of DLBCL is fast and cost-effective but suffers from a relevant degree of discordance between algorithms and does not accurately reflect GEP assays [14]. To overcome the need for fresh-frozen material and provide a routinely applicable, accurate test for ABC/GCB subtyping, the Nanostring nCounter-based Lymph2Cx assay was successfully developed and tested [15, 16]. The test was also used in DLBCL clinical trials [17–19], but only few studies reported the day-to-day experience in pathology laboratories [20–23].

We prospectively tested 147 aggressive B-cell lymphoma samples in a routine diagnostic setting within the Reference Center for Lymph Node Pathology and Hematopoietic Diseases of the Institute of Pathology of the University of Würzburg, Germany. In this report, we describe our experience with the routine implementation of the Lymph2Cx assay and describe the major technical and diagnostic pitfalls as well as the discrepancies in comparison with the results of the Hans algorithm and the correlation with the fluorescence in situ hybridization (FISH) results.

## Materials and methods

### Case series

**Fig. 1:**
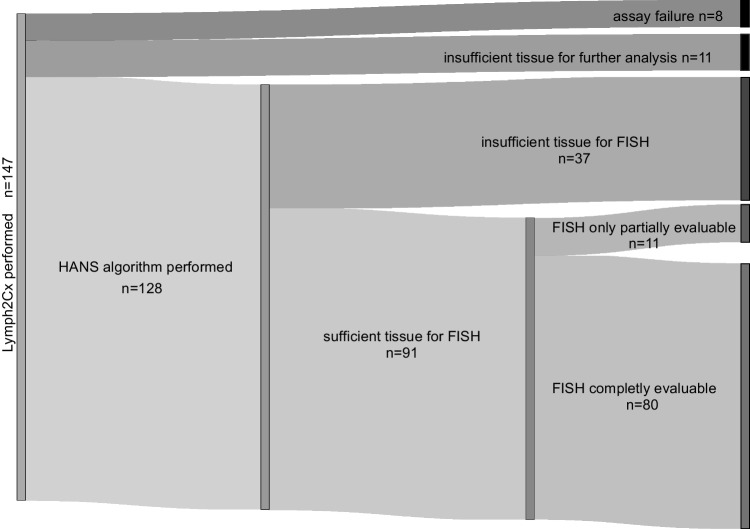
Case series. Sankey flow diagram visualizes cohort selection, excluded samples and reasons for exclusion

Cases were tested between 2016 and 2019 and were routinely diagnosed as DLBCL according to the current WHO criteria [12], four cases were eventually classified as high grade B-cell lymphomas (HGBCL-DH/TH) after FISH analysis. We selected cases for our study cohort based on tumor cell content (> 60%) and needle biopsies were excluded beforehand. Cohort selection and excluded samples are visualized in Fig. 1. Table 1 includes the basic patient characteristics. Unfortunately, no further clinical data or follow-up data were available. The cases are part of the biobank at the Institute of Pathology of the University of Würzburg, Germany, and were used according to the ethical guidelines of the Medical Faculty.

**Table 1:** Patient cohort

			Cohort of 128 n or median (%, range)	Cohort of 91 n or median (%, range)
Male			81 (63.3)	56 (61.5)
Female			47 (36.7)	35 (38.5)
Age			72 (94–17)	74 (93–17)
Hans algorithm	Non-GCB		71 (55.5)	48 (52.7)
	GCB		57 (44.5)	43 (47.3)
Lymph2Cx	ABC		74 (57.8)	53 (58.2)
	GCB		47 (36.7)	33 (36.3)
	Unclassified		7 (5.5)	5 (5.5)
FISH	MYC	+		10 (11.0)
		−		71 (78.0)
		n a		10 (11.0)
	BCL2	+		10 (11.0)
		−		75 (82.4)
		n a		6 (6.6)
	BCL6	+		27 (29.7)
		−		62 (68.1)
		n a		2 (2.2)

### Immunohistochemistry

Immunohistochemistry (IHC) was performed according to standard protocols using the following antibodies: CD10 (clone 56C6, Leica Novocastra, Nußloch, Germany), MUM1/IRF4 (clone MUM1p, Agilent Dako, Waldbronn, Germany), BCL6 (clone PG-B6p, Agilent Dako). Detailed IHC information was not available for 18/128 cases, but all cases were classified according to the Hans algorithm as described [13]. Central pathology revision and IHC evaluation was conducted by three expert hematopathologists (AZ, IA, and AR). Cases with sufficient residual tissue after IHC (> 60% tumor cell content) were selected for the Lymph2Cx and, whenever possible, for FISH assays.

### Lymph2Cx assay

The Lymph2Cx assay was performed as previously described [15]. Briefly, RNA from 147 samples was extracted with the AllPrep DNA/RNA Mini Kit (Qiagen, Hilden Germany). The eluted RNA quality and concentration were measured with the NanoDrop spectrophotometer (Thermo Fisher Scientific, Waltham, MA, USA). The RNA was collected and the NanoString Lymph2Cx Assay (NanoString Technologies) was performed in batches of 12 cases according to the NanoString protocol, as previously published [15]. No RNA quality criteria was applied and 400 ng total RNA was used. The assay was run on a Generation 1 nCounter platform (NanoString Technologies) and was analyzed by one of the authors (DS) according to the previously published algorithm [15].

### FISH and ISH

FISH assays for *MYC*, *BCL6*, *BCL2 and IRF4/DUSP22* (6p25) were performed either on whole slides or on tissue microarrays (1 mm core diameter in duplicate) according to standard protocols. The following ZytoLight Spec dual-color break-apart probes from ZytoVision GmbH (Bremerhaven, Germany) were used: MYC (Z-2090–50), BCL6 (Z-2177–50), and BCL2 (Z-2192–50). The *IRF4/DUSP22* (KBI-10613) probe was from Kreatech Biotechnology B.V. (Amsterdam, The Netherlands) and purchased via Leica Biosystems (Wetzlar, Germany). Slides were counterstained with DAPI and evaluated with a fluorescence microscope (Zeiss). Cut-offs for positivity were defined from averaged negative controls (reactive tonsils) and were 7% (*BCL2*), 9% (*BCL6*) 7% (*MYC*) and 14% (*IRF4/DUSP22*). For Epstein-Barr Virus-Encoded RNA (EBER) detection, ISH was performed on whole slides or on tissue microarrays by use of the Ventana ready-to-use kit, according to the appropriate protocols, within an automated immunostainer (Benchmark XT; Ventana/Roche,Tucson, AZ, USA).

### Statistical analysis

Statistical analysis was performed with standard *t*-test using Excel (Microsoft, Redmond, WA, USA). Continuous variables are summarized with descriptive statistics, such as mean/median, and range. Categorical variables are displayed with frequency and percentage.

## Results

### Patient cohort and characteristics

Of the 147 initially included FFPE samples, 19 cases were excluded (8 due to Lymph2Cx assay failure or poor data quality, 1 due to depleted tumor tissue and 10 due to incomplete information and material (Fig. 1). Eventually, we could evaluate 128 samples using the Lymph2Cx algorithm. This cohort included samples from 81 male and 47 female patients and the median age was 72 years. Of these 128 samples, 57 were classified as GCB and 71 as non-GCB according to the Hans algorithm. The Lymph2Cx assay cell of origin (COO) assignments of these 128 cases were 47 GCB, 74 ABC and 7 remained unclassified (Table 1). For 91 cases, enough residual tissue was left for further FISH analyses (Fig. 1), and this cohort consisted of 56 samples from male and 35 female patients with a median age of 74 years. Here, the COO distribution was very similar, as the Hans algorithm classified 43 cases as GCB and 48 as non-GCB, whereas the Lymph2Cx assay classified 33 cases as GCB, 53 as ABC and five as unclassified (Online Resource 1, Table 1).

Comparing results with the classification provided by the Hans algorithm in our cohort of 128 cases, we obtained an absolute concordance of 82% (86.8% after excluding seven unclassified cases) (Online Resource 2, Fig. 2). Discrepant results were obtained in 18% (13.2% after excluding unclassified cases), including five cases classified as GCB by the Lymph2Cx assay and as non-GCB by the Hans algorithm, 11 lymphomas classified as ABC by the Lymph2Cx assay and as GCB by the Hans algorithm and seven cases unclassified by the Lymph2Cx assay, of which four were classified as GCB and three as non-GCB by the Hans algorithm (Fig. 3). In the subgroup with additional FISH analysis (n = 91), the distribution was very similar; Lymph2Cx and Hans algorithm showed a concordance of 82.4% (87.2% after excluding five unclassified cases). Here, two cases were classified as GCB by the Lymph2Cx assay and as non-GCB by the Hans algorithm, nine cases were categorized as ABC by the Lymph2Cx assay and as GCB by the Hans algorithm and five cases were unclassified by the Lymph2Cx assay, of which three were designated as GCB and two as non-GCB by the Hans algorithm (Online Resource 2 and Fig. 3). In both cohorts, the discordant cases showed a Lymph2Cx score and calculated ABC likelihood predominantly in-between the concordant GCB and ABC groups tending towards ABC (Fig. 4a–d). Additionally, we tested our cases where possible for EBER and out of 85 evaluable cases, only one was positive, a *MYC* translocated, concordant GCB-type (Online resource 2).

**Fig. 2:**
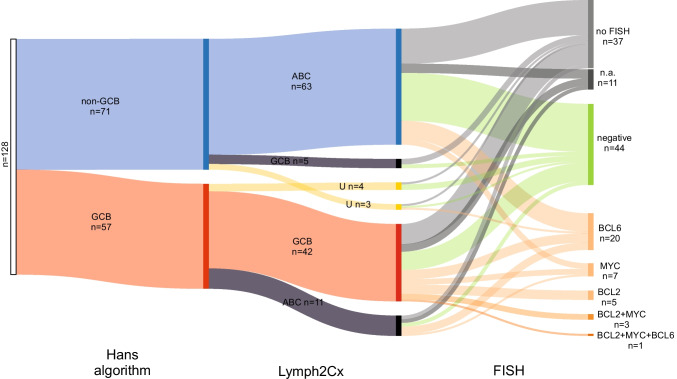
Sankey diagram mapping the COO subclassification by Hans algorithm and Lymph2Cx in combination with FISH data for *BCL6*, *BCL2* and *MYC* translocations

**Fig. 3:**
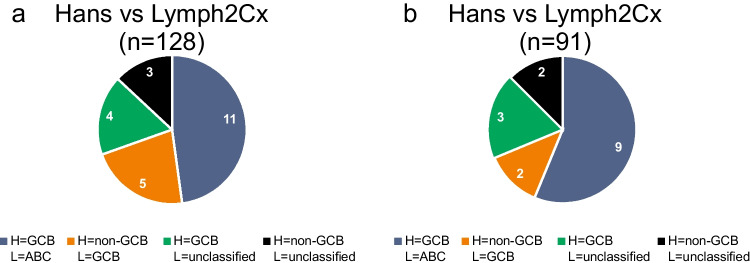
In both cohorts ((**a**) including all cases with data from Lymph2Cx and Hans classifier available, n = 128 and (**b**) including all cases with additional FISH data available, n = 91) the allocation of discrepancies is alike. H (Hans algorithm), L (Lymph2Cx), GCB (germinal center B-cell), ABC (activated B-cell)

**Fig. 4:**
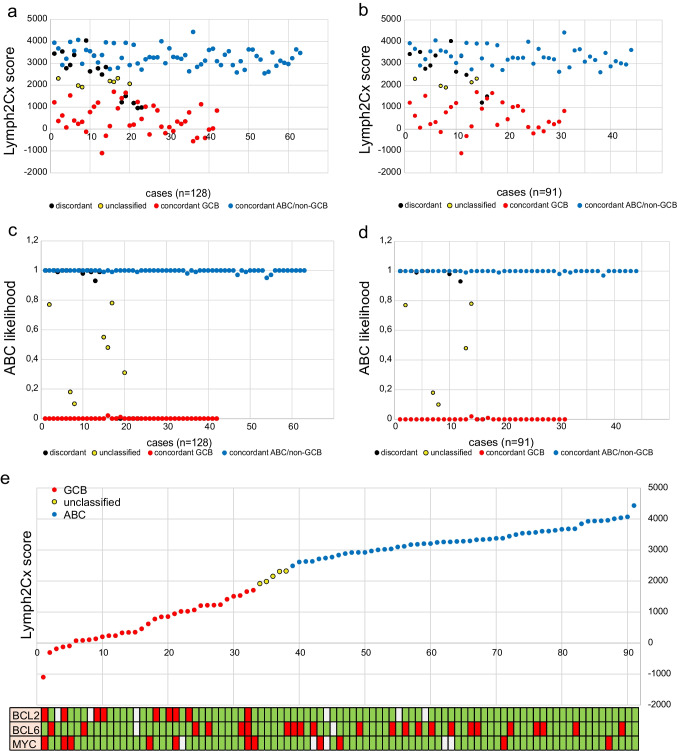
Distribution of cases according to Lymph2Cx score, ABC likelihood and genetic rearrangements. **a** and (**b**) Illustrate the Lymph2Cx score distribution and (**c**) and (**d**) the calculated ABC likelihood in the two cohorts a, c n = 128, b,d n = 91. Cases are sorted according to the subclassification group by Hans algorithm, concordance to Lymph2Cx and consecutive number. **e** Depicts the spreading of *BCL2*, *BCL6* and *MYC* rearrangements in correlation to the Lymph2Cx score

A review of discordant cases showed that the main cause of the discrepancy is the intrinsic lack of the “unclassified” category in the Hans algorithm, with 7/23 (30.4%) discrepant cases falling within this category. Detailed IHC was available for 19/23 cases, all of which were BCL6-positive, and seven discrepant cases showed expression of CD10 and were therefore classified as GCB according to the Hans algorithm. However, Lymph2Cx classified them as either ABC (four) or unclassified (three) (Online Resource 2). Four of these (three unclassified and one ABC) contained large numbers of T-cells, two were CD5-positive (both ABC) and one had an immunoblastic/plasmablastic morphology (ABC) (Online Resource 2 and data not shown). Considering the possible reasons for the discrepancy in all 23 cases, we saw very abundant T-cells (six cases, five of them GCB by IHC and ABC or unclassified by Lymph2Cx), poor fixation (three cases), expression of CD5 in neoplastic cells (three cases, all GCB by IHC and ABC by Lymph2Cx), anaplastic, immunoblastic/plasmablastic morphology (two cases, both GCB by IHC and ABC by Lymph2Cx). Additionally, we checked in the cohort of discrepant cases the IRF4 IHC positive cases for *IRF4/DUSP22* break by FISH. Out of these, we had material for *IRF4/DUSP22* FISH for 8 cases and 7 were evaluable. Out of these 7, two were positive, one non-GCB by Hans and unclassified by Lymph2Cx and one case being GCB by Hans and ABC by Lymph2Cx. Of the 7 evaluable cases, 3 were triple positive by IHC and only one was *IRF4/DUSP22* FISH positive (Online Resource 2). Other possible reasons for discrepancy could be subvital tumor cells/necrotic tissue (one case), poor fixation (one case) and for five cases, we detected no apparent reason. Additionally, two discrepant cases were testicular DLBCL (both GCB by IHC and ABC by Lymph2Cx) and overall 10/23 cases were extranodal lymphoma infiltrates (Online Resource 3).

Of all cases with complete *MYC*, *BCL2* and *BCL6* FISH results (80/91, 87.9%), 11 showed a *MYC* rearrangement (of which three were in combination with *BCL2* and one triple-hit), nine a *BCL2* break (three in combination with *MYC*, one as a triple hit) and 21 a *BCL6* break (one as triple-hit). Comparing the Lymph2Cx score and categories, we observed a robust clustering of *BCL2* rearrangements in the GCB group (9/9, 100%). In addition, *MYC* breaks tended to be more frequent in the evaluable GCB group (7/32, 21.9%) against 4/49 (8.2%) in the ABC group. Meanwhile, the distribution of *BCL6* breaks was more scattered but with a slight prevalence in the ABC subgroup (16/23, 69.6% versus 6/23 26.1% in the GCB group). One case with *BCL6* break was unclassified by Lymph2Cx. As a result, the one triple and all three double-hit HGBCL-DH/TH cases of our series were in the GCB group (Online Resource 2, Fig. 2 and Fig. 4e).

## Discussion

The distinction between ABC- and GCB-type DLBCL has relevant prognostic and therapeutic implications and has become mandatory in the 2017 WHO classification of lymphoid neoplasms and will continue to play a role in the upcoming 5^th^ edition 2022. The Lymph2Cx assay was proposed as a user-friendly assay that provides reliable result using FFPE material [15] to identify ABC- and GCB-type DLBCL. It was used in clinical trials [17–19], but reports about its use in the routine practice of pathology have been limited to five Asian series [20, 21, 24–26], and two Western series [22, 23]. Online Resource 4 summarizes the published data of these series. This literature comparison highlights that only one of these studies included FISH analysis. Thus, our study adds profound data for the comparison of the Hans algorithm with the Lymph2Cx assay in context with FISH results.

We report here our real-world experience with the prospective application of the Lymph2Cx in a routine setting from a large European reference center. In comparison to other studies [15, 16, 18, 23], the distribution of ABC and GCB in this cohort showed a slight dominance of ABC cases. A reason for this difference could be a potential selection bias of a reference center, or an increased number of extranodal cases in our cohort. However, five Asian studies also report an ABC-type dominance (Online Resource 4) [20, 21, 24–26]. We focused on reporting the discrepancies with the most widely used IHC algorithm (Hans) as well as the correlation with the FISH findings regarding the status of *MYC*, *BCL6*, and *BCL2*.

Our data show that, as previously reported, the Hans algorithm is suboptimal for the subclassification of DLBCL in GCB and non-GCB groups, although our concordance (82%) was slightly higher than what has been reported in previous real-world series (range 69–79%), possibly because of the relatively low number of cases falling in the unclassified category (7/128, 5%). After excluding unclassified cases, our series shows a concordance rate of 87% (range from previous series 81–95%), possibly reflecting the improved algorithm evaluation through a high volume of diagnostic cases in our reference center [20–26]. Another possible explanation for our relatively high concordance rate is the exclusion of needle biopsies, which are often difficult to evaluate due to limited material as well as crush and fixation artifacts, which might lead to misinterpretation of IHC results. A third possible reason is the selection of cases with more than 60% tumor cell content, thereby limiting gene expression artifacts due to a large number of contaminating bystander (usually T-) cells.

We found discrepancies between the Hans algorithm and the Lymph2Cx assay in 23 cases (23/128, 18%), including seven cases left unclassified by the Lymph2Cx assay. Four discrepant cases were classified as GCB because of CD10 expression, but were reclassified as ABC using the Lymph2Cx assay. Two of these were also CD5-positive, in concordance with the fact that > 90% of CD5-positive DLBCL belong to the ABC group [27]. Although rare, a co-expression of CD5 in DLBCL classified as GCB by the Hans algorithm might indicate that these cases belong biologically rather to the ABC category, and should probably be classified accordingly. One misclassified CD10-positive case showed a plasmablastic/immunoblastic morphology (but was classified as DLBCL because of strong CD20 expression); it might be hypothesized that this case might be biologically close to plasmablastic lymphoma, which express CD10 with relatively high frequency (20–40%) [28]. Some of the genes implemented in the Lymph2Cx signature are also expressed in T-cells, like *IRF4*, *PIM2* and *MYBL1* [29–32] and four misclassified CD10-positive cases displayed a large number of T-cells, which might be a confounding factor for technologies, like Lymph2Cx, which are based on an average gene expression profile. Without the possibility to analyze purified tumor cells, it is debatable whether in such cases IHC or gene expression studies might be more accurate, since IHC has the ability to profile also a small number of morphologically atypical elements within a large number of accompanying non-neoplastic cells. Our decision to select cases with a tumor cell content > 60% seems therefore a reasonable approach to limit such discrepant findings. Two discrepant cases were testicular DLBCL, both were classified as GCB (by BCL6-only phenotype) by IHC and as ABC by the Lymph2Cx assay; in accordance with the literature, 11/12 (92%) of our testicular cases were classified as ABC by the Lymph2Cx assay, the only one case classified as GCB being also CD10-positive. These data might suggest that future algorithms should include CD5 and localization in their variables. The new WHO and ICC recommendations deal with this topic and now describe more distinct entities, including a group of ‘immune-privileged lymphomas’ [33, 34].

The analysis of FISH results showed that, as expected from the published literature, *BCL2* rearrangements (either alone or as double/triple hits) clustered under the umbrella of GCB (classified by Lymph2Cx), so that the presence of a *BLC2* break is, in our series, a highly (100%) specific, but not very sensitive (30%) marker of aggressive B-cell lymphomas of GCB-type. However, upfront use of FISH is not necessary since the Hans algorithm did not misclassify any of the *BCL2* translocated cases. Among the 23 misclassified cases, we found a surprising enrichment for *BCL6* translocations within cases classified as GCB by Hans and as ABC by Lymph2Cx, with five of the eight evaluable showing a *BCL6* break (Online Resource 2). This finding is in line with our previous data [35, 36], showing that follicular lymphomas lacking the *BCL2* translocation show a late germinal center (GC) phenotype and from other groups [37] showing that these lymphomas transform in, about half of the cases, to ABC DLBCL, and a portion of these cases bear a *BCL6* translocation. It is possible that these transformed FL could still bear a partial or complete GC phenotype, although showing an ABC expression profile, in analogy with t(14;18)-negative FL [35, 36]. All in all, among 22 *BCL6*-break-positive cases (excluding one HGBCL-TH), 16 cases were ABC, one unclassified, and five GCB cases using the Lymph2Cx assay. Since four of these GCB cases were CD10-positive, an improved version of a novel algorithm might also include a break-apart FISH for *BCL6*, since practically all CD10 negative cases with a *BCL6*-break were ABC. The additional FISH analysis for IRF4/DUPS22 showed in the small subgroup of samples neither triple positive IHC nor IRF4 IHC positivity as a main cause for discrepancy between Hans algorithm and Lymph2Cx assay or as indication for an *IRF4* translocation.

The main limiting factor for applying the Lymph2Cx test in our series was the availability of sufficient residual tumor tissue after IHC was performed for diagnostic purposes. This obstacle could be partly overcome by ordering the test in parallel with IHC, in order to spare precious tissue when using core biopsy material or pre-cutting paraffin rolls for molecular pathology.

RNA-based assays are more sensitive to pre-analytical variables as well as to the extraction methodology and post-extraction handling. However, we obtained RNA of sufficient quantity and quality by routinely used methods in 128/147 samples, showing a high success rate, which is probably mainly achieved because no extensive sample handling is required and the technical advantage of using short hybridization probes, which provide robust results with partially degraded samples. Our lab procedures allowed one Lymph2Cx assay run for 12 collected cases, with a sample-to-result time of 4 days with approximately 13 h hands-on time including cutting and RNA extraction. The diagnostic findings, according to the Lymph2Cx assay, were generally sent as follow-up report a few days later than the IHC-based report. Currently, the Lymph2Cx assay is available at NanoString as Lymphoma Subtyping Test (LST) for research use only and the collected data is analyzed by NanoString and reported back. The test is CE-IVD marked and manufactured by NanoString under contract on behalf of Veracyte. It has not been cleared nor approved by the U.S. Food and Drug Administration. It is currently available for companion diagnostic clinical studies and is not commercially available.

In conclusion, the Lymph2Cx provided accurate, reliable results in the routine clinical setting for DLBCL diagnosis and can be implemented in the day-to-day practice of hematopathology laboratories. In view of a more personalized approach to lymphoma therapy in the near future, which might include an extensive molecular work-up, this assay might become a routinely used test in the characterization of aggressive B-cell lymphoma alongside IHC, FISH, clonality analysis and mutation detection. However, these analyses are relatively costly and require a well-equipped laboratory, two factors that might hamper a widespread application.

## Supplementary Information

Below is the link to the electronic supplementary material.

Supplementary file1 (PDF 1381 KB)

Supplementary file2 (XLSX 25 KB)

Supplementary file3 (PDF 1371 KB)

Supplementary file4 (XLSX 13 KB)
