# Supplementary material for: Routine application of the Lymph2Cx assay for the subclassification of aggressive B-cell lymphoma: report of a prospective real-world series
Source: Virchows Arch. 2022 Oct 11;481(6):935–43. doi: 10.1007/s00428-022-03420-6 (PMC9734243; doi:10.1007/s00428-022-03420-6)
Supplement: Supplementary file 3 — Supplementary file3 (PDF 1371 KB) [file 428_2022_3420_MOESM3_ESM.pdf]

Online Resource 3

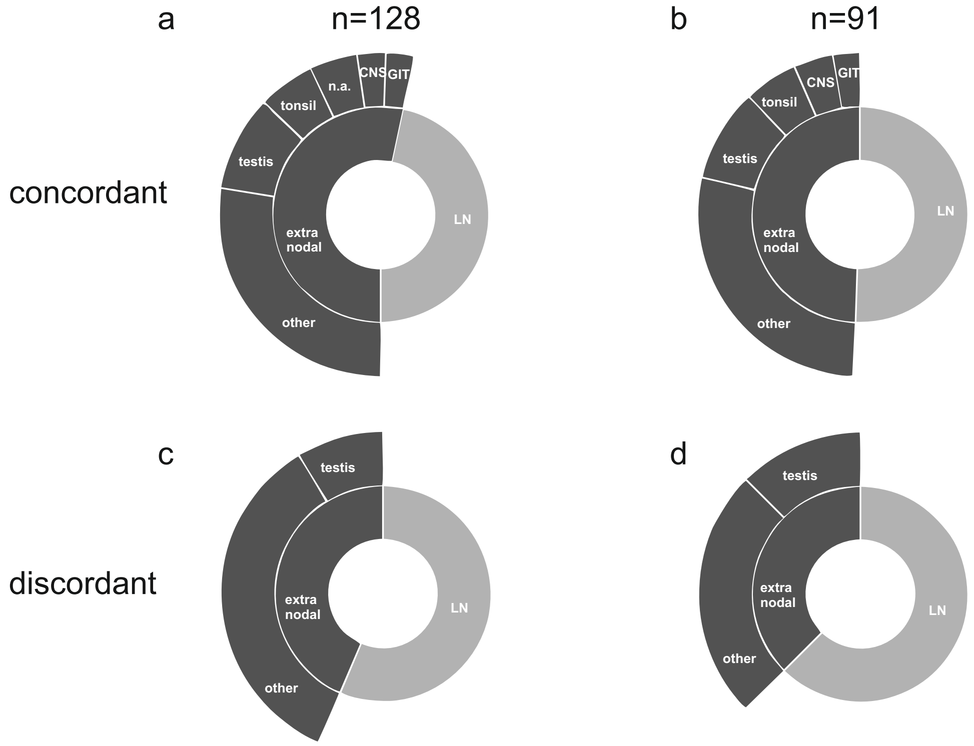

Routine application of the Lymph2Cx Assay for the subclassification of aggressive B-cell lymphoma: report of a prospective real-world series.

Alberto Zamò, Elena Gerhard-Hartmann, German Ott, Ioannis Anagnostopoulos, David W. Scott, Andreas Rosenwald, Hilka Rauert-Wunderlich

Corresponding author:

hilka.rauert-wunderlich@uni-wuerzburg.de

Virchows Archiv
